# Methods of nutrition surveillance in low-income countries

**DOI:** 10.1186/s12982-016-0045-z

**Published:** 2016-03-18

**Authors:** Veronica Tuffrey, Andrew Hall

**Affiliations:** Faculty of Science and Technology, University of Westminster, 115 New Cavendish Street, London, W1W 6UW UK; Save the Children, 1 St John’s Lane, London, EC1M 4AR UK

**Keywords:** Anthropometry, Nutrition assessment, Public health, Program evaluation, Monitoring, Surveillance, Surveys, Timely warning, Malnutrition, Low-income

## Abstract

**Background:**

In 1974 a joint FAO/UNICEF/WHO Expert Committee met to develop methods for nutrition surveillance. There has been much interest and activity in this topic since then, however there is a lack of guidance for practitioners and confusion exists around the terminology of nutrition surveillance. In this paper we propose a classification of data collection activities, consider the technical issues for each category, and examine the potential applications and challenges related to information and communication technology.

**Analysis:**

There are three major approaches used to collect primary data for nutrition surveillance: repeated cross-sectional surveys; community-based sentinel monitoring; and the collection of data in schools. There are three major sources of secondary data for surveillance: from feeding centres, health facilities, and community-based data collection, including mass screening for malnutrition in children. Surveillance systems involving repeated surveys are suitable for monitoring and comparing national trends and for planning and policy development. To plan at a local level, surveys at district level or in programme implementation areas are ideal, but given the usually high cost of primary data collection, data obtained from health systems are more appropriate provided they are interpreted with caution and with contextual information. For early warning, data from health systems and sentinel site assessments may be valuable, if consistent in their methods of collection and any systematic bias is deemed to be steady. For evaluation purposes, surveillance systems can only give plausible evidence of whether a programme is effective. However the implementation of programmes can be monitored as long as data are collected on process indicators such as access to, and use of, services. Surveillance systems also have an important role to provide information that can be used for advocacy and for promoting accountability for actions or lack of actions, including service delivery.

**Conclusion:**

This paper identifies issues that affect the collection of nutrition surveillance data, and proposes definitions of terms to differentiate between diverse sources of data of variable accuracy and validity. Increased interest in nutrition globally has resulted in high level commitments to reduce and prevent undernutrition. This review helps to address the need for accurate and regular data to convert these commitments into practice.

## Background

There is currently substantial interest in nutrition, evident from political and financial commitments made by national governments, international organisations and donors, however action to convert these commitments into practice is being hindered by a lack of data [1]. More and effective surveillance of the nutrition situation in countries to the level of districts is needed to support national policy development and local programme planning, and to provide timely warning of shocks. Also data are needed to track progress towards new goals and targets. To help address these needs for nutrition-relevant data, this paper reviews the sources of nutrition surveillance data, and how they are collected.

### Definition and history of nutrition surveillance

Nutrition surveillance in low-income countries involves the regular and systematic collection of data on nutritional outcomes and exposures, as specified in 1976 in the first guidance on the subject: “Surveillance should provide ongoing information about the nutritional conditions of the population and the factors that influence them” [2]. Information derived from nutrition surveillance has been used in several ways: to monitor the nutrition situation; to identify factors associated with malnutrition; to inform nutrition policies and programmes; to track progress towards achieving nutrition goals; to serve as an early warning of increased nutritional risk; to assess the delivery and coverage of services; to evaluate programmes and interventions; and to detect the impact of changes in policies. The issues relating to the sustainability of nutrition surveillance activities are considered in another paper [3], whilst in this paper the methods used to collect data are reviewed.

The foundations of nutrition surveillance were laid in 1963 when the term ‘surveillance’ was defined by Langmuir in relation to monitoring trends in disease in the population, rather than monitoring individuals at risk of disease [4]. At the first World Food Conference in 1974, the FAO, the WHO and UNICEF were invited to establish a global nutrition surveillance system. The methods were then developed by an expert committee [2] and this was followed by a period of considerable activity to set up national surveillance systems. By the early 1980s there were nutrition surveillance systems in about 20 countries. Further guidance was published in 1984 which provided the definition of nutrition surveillance that is most often quoted: “… to watch over nutrition, in order to make decisions which lead to improvements in nutrition in populations” [5]. These early surveillance systems were primarily based on growth monitoring data from clinics plus infrequent surveys, while a few school census systems existed, mainly in Central America. Some systems that started at this time have lasted and were useful, but most eventually ceased as they used complex and expensive methods to collect data which generated information so slowly that it was of little value [6]. It was recently estimated that there are 31 national surveillance systems administered under the auspices of government public health authorities [7].

There is only a small body of literature published on nutrition surveillance in low-income countries. Before the mid-1990s, the papers and reports relate mainly to guidance around setting up surveillance systems [for example, 8], with a few descriptions of their design and implementation [or example, 9]. From the mid-1990s onwards and particularly since 2000, the literature mainly concerns the analysis of surveillance data to address public health issues of international significance such as micronutrient deficiencies, the double burden of malnutrition, and links between anthropometric status and exposures including tobacco use, education, expenditure on food and economic crises [for example, 10–12]. During the 1980s several reviews were published of surveillance methods and activities, but they have been infrequent since then, averaging one per decade [6, 13, 14] with the most recent of these relating to surveillance in humanitarian settings [14]. Updated guidance for national systems was recently published by the WHO Regional Office for the Eastern Mediterranean [15], and the US Food and Nutrition Technical Assistance Project produced an inventory of systems run by government public health authorities [7].

Given the recently increased understanding and recognition of the harmful consequences of undernutrition for individuals, communities and nations, a review of the methods used for nutrition surveillance is timely. As well as increasing the risk of dying [16], undernutrition also impairs children’s cognitive development and restricts their physical growth which, in turn, delays enrolment in school and affects educational outcomes and earnings in adulthood [17]. The two high-profile series of papers in The Lancet journal in 2008 and 2013 have helped to draw attention to the need to prevent or reduce malnutrition early in children’s lives, and to the cost-effectiveness of relatively simple interventions to do this. A lack of consistently collected data on important indicators is holding back actions to address poor nutrition [1, 18] so improving the processes of nutrition surveillance could help to redress this lack.

There is currently substantial political momentum to reduce the numbers of children affected by undernutrition, as demonstrated by the political and financial commitments made by national governments, international organisations, and donors. For example, the World Health Assembly (WHA) adopted a Comprehensive Implementation Plan on Maternal, Infant and Young Child Nutrition in May 2012. They agreed to commit to a number of targets to be achieved by 2025, known as the WHA global targets, including a reduction by 40 % in the number of stunted children in the world and a reduction of the prevalence of wasting to 5 % [19]. These targets were integrated into the second of the post-2015 Sustainable Development Goals [20]. At the Nutrition for Growth summit in London, a set of individual commitments to beat hunger and improve nutrition were made in 2013, including a $4.15 billion financial commitment to scale up nutrition specific actions by 2020 [21]. Many countries recently signed up to the Declaration of the Second International Conference on Nutrition and committed themselves to taking action on several fronts [22]. Existing sources of data are insufficient to help allocate the funds needed for nutrition initiatives and to track progress towards these goals [23], so better nutrition surveillance systems would help to rectify this.

Another reason to review methods for nutrition surveillance now is the need to predict and detect shocks in regions of the world where the combination of climate change, rapid population growth, conflict, and food price volatility has resulted in almost permanent crisis. This environment, within which reside much of the world’s chronically poor and malnourished population, is increasingly unstable, and resilience to shocks and stresses due to natural and man-made disasters needs both to be strengthened and regularly assessed [24].

This paper reviews methods of nutrition surveillance in low-income countries. The structure of the paper is as follows: we describe current approaches applied to data collection with their pros and cons; we outline the things to consider when choosing between approaches, including context and objectives; we examine issues related to the collation, analysis and interpretation of data; we explore potential applications and challenges in relation to recent developments in electronic technology; and finally we propose definitions of terms to maximise clarity in discussions and help achieve consistency between activities in different locations. Together with our paper which addresses the institutional issues [3], the aim of this paper is to aid practitioners and agencies to amend or design cost-effective and sustainable nutrition surveillance activities in order to prevent poor nutrition in low-income countries.

## Analysis

### Outline of current approaches

Nutrition surveillance is a process of monitoring trends in the nutrition situation over time to inform decision-making. It does not necessarily trigger action, rather it informs decisions about when actions are needed and guides the choice of actions, such as making or amending policies, introducing a programme, or changing an existing programme. For these purposes, data relating to both nutritional outcomes and exposures need to be collected systematically, in which systematic refers both to the regularity of data collection and the consistent use of trusted methods.

**Fig. 1:**
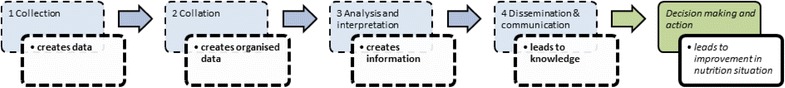
The process of nutrition surveillance, and the products of each stage

Figure 1 shows the four stages of the process of surveillance, and the products of each stage. Data are defined here as simple measures or characteristics of people and things, and have little inherent meaning or value until aggregated. After the analysis and interpretation of data, patterns can be identified, thereby creating information. Finally, the use of information to generate recommendations, rules for action, and changes in behaviour signifies the creation of knowledge that is used to make decisions [25]. Figure 1 illustrates how organised data result from the first two stages of the surveillance process, information is created from the third, and knowledge is derived from the final stage. The figure also shows how knowledge gained from surveillance should contribute to making decisions to improve nutrition. The utility of the system depends on the extent to which the information it yields is used effectively [26].

It is useful at the outset to distinguish between ‘nutrition surveillance systems’ and ‘nutrition information systems’. In everyday language an ‘information system’ refers to an integrated set of hardware, software, data, people and procedures that produces information. We propose that the term ‘nutrition surveillance system’ is reserved for systems in which original nutrition data are collected regularly. Thus, in the same way that health surveillance is one component of health information systems [27], nutrition surveillance can be considered as one component of nutrition information systems. For example, repeated surveys at six-monthly intervals in Ethiopia provide a system of nutrition surveillance that contributes to an information system administered by the Ethiopian Emergency Nutrition Coordination Unit [28]. Similarly, the recently published guidance [15] pertains to national information systems.

As will become clear from the discussion in the remainder of the paper, data used for nutrition surveillance do not fall into neat categories, so there exists a “lack of clarity in defining, classifying, and describing different methodological approaches to nutrition surveillance” [14]. In this paper we propose a classification for the data collection activities, to aid dialogue and planning. The existing lack of clarity partly relates to the use of health systems as a source of surveillance data. Such data have been described both as “administrative data” [29] and “secondary data” [15]. These terms are considered as synonyms in the context of official collections of data, and administrative sources are defined as “data holdings containing information which is not primarily collected for statistical purposes” [30]. In the context of nutrition in low-income countries, anthropometric data collected through health systems are sometimes recorded by staff principally for statistical purposes rather than for diagnostic reasons, such as data from growth monitoring. In these situations such data should strictly be classified as primary but they are not representative because the children brought to health facilities are a selected sample, like other types of data from health systems, such as indicators of service delivery, which are conventionally classified as secondary or administrative data. Therefore we classify original data that are collected exclusively for surveillance purposes as ‘primary’ while for data collected for any other reason we use the term ‘secondary’. All data, whether primary and original or secondary and administrative, need to be of high quality and internally valid; primary data should be externally valid, too.

A recent review identified five approaches to collecting anthropometric data for surveillance in humanitarian settings [14]. The present review encompasses the context of development as well as humanitarian circumstances, so additional methods are included here. A description of the methods is provided only where they have not been addressed in the previous review [14]. The six approaches are here classified into two categories according to whether they depend on primary or secondary data, as defined above. Primary data includes the first two approaches described previously, repeated cross-sectional surveys and data collection at sentinel sites in communities [14], together with one more approach, that of collecting data about children attending schools. Secondary data includes the remaining three approaches described in the previous review: the use of admissions data from feeding centres; data collected at health facilities; and data collected in the community, termed “mass screenings” in the prior review [14].

### Collection of primary data for nutrition surveillance

Strategies to collect primary data for surveillance are distinguished from those for secondary data by their sampling methods, most simply described as selection based on probability, rather than self-selected and non-random. The sites where primary data are collected may be sampled randomly or repeatedly, depending on the system chosen.

In a sentinel surveillance system, data are regularly reported from a specified sample of sites, often using purposive sampling at some stage, which can be used to indicate trends within the target population. The target population can be national, such as for the system in Mozambique [31], or living in an area which is highly vulnerable to malnutrition, a livelihood zone or some other defined geographical area. When properly implemented, these systems offer an effective method of surveillance using limited resources, and enable prompt and flexible investigation and then monitoring of a suspected problem [32].

Surveys entirely using probability-based sampling methods can be used for surveillance if they are regularly repeated. As long as the sampling methods are applied properly, surveys have the potential to provide data that are representative of the target population compared with repeated assessments using purposive sampling. Most surveys in low-income countries use stratified, multistage cluster designs. This avoids the need for a complete list of subjects in the population, which would be necessary for a purely random sample, so once the clusters have been randomly selected, perhaps with a probability that is proportionate to their population, a list of subjects needs only to be compiled for the selected sample of clusters and the sample of subjects can then be drawn from those lists. This helps to simplify the process of sampling and reduce the cost of data collection. The process has been simplified further by selecting subjects from households using a transect through a cluster, although this may lead to a biased estimate of the indicator of interest [33]. Cluster surveys were first used to assess immunization coverage in developing countries [34] and have been validated for estimating nutritional status [35].

#### Primary data collection: cross-sectional surveys

##### Large scale nationally representative surveys

Nationally representative data for many low-income countries are provided by two programmes of household surveys: the Demographic and Health Surveys (DHS) supported by USAID, and the Multiple Indicator Cluster Surveys (MICS) led by UNICEF. These programmes have provided valuable data to assess trends in nutrition nationally and globally since the DHS project started in the mid-1980s and MICS in the mid-1990s, and they have helped to monitor progress towards targets such as the Millennium Development Goals (MDGs) [36]. In terms of national surveillance, since there is generally a survey of one of these two types every 3 years or so, their findings enable long-term trends to be observed [29] and may provide a means of verifying the findings of other, more frequent sources of data. Methods are standardised, but there are challenges related to quality-control due to the need to train and supervise hundreds of surveyors. In any nutrition survey a large amount of random error in measurements leads to a larger standard deviation of Z-scores than due to biological variation [37] which in turn leads to an overestimate of the prevalence of undernutrition [38, 39]. Many DHS surveys have standard deviations greatly exceeding the quality criteria defined by the World Health Organization [37]. Also such surveys are expensive, and it usually takes at least a year for the findings to be released after data collection. Generally a sample of around 15,000 children and their households is included in a DHS and around 10,000 in a MICS. The large sample sizes enable statistically reliable estimates of most nutritional indicators to be made at national, urban–rural, and regional levels, but not at lower administrative levels such as districts [36], for which there is a growing demand for data as services are decentralised.

Given the delay between collecting data and producing validated findings, these surveys are not suitable for tracking the prevalence of wasting near or above emergency thresholds. So in West and Central Africa, UNICEF has supported around 15 national-level nutrition surveys each year since 2008 using Standardized Monitoring and Assessment of Relief and Transition methods (SMART—a well-accepted collection of best practices for the implementation of nutrition surveys [40, 41]). It is recognized that conditions need to be tracked regularly throughout an entire country, not just in areas perceived to be at highest risk. For example in the Democratic Republic of the Congo, nutrition conditions were closely followed in the war-affected eastern regions but when surveys were finally conducted in the southern half of the country, it was found that the prevalence of wasting was much higher in the regions not affected by violence than in the areas considered to be experiencing an emergency (personal communication, Robert Johnston).

##### Repeated sub-national cross-sectional surveys

Repeated surveys using probability sampling methods at sub-national level for the purposes of nutrition surveillance are undertaken at intervals of 1, 3, 4, 6 or 12 months. Cluster sampling is used for practical reasons as described above, and new clusters may be drawn for each round of data collection, or clusters drawn initially may be used in subsequent rounds [14].

The sampling design of 30 × 7 (clusters × children) was initially used to estimate vaccination coverage [34], and later the sampling design of 30 × 30 (clusters × children) was recommended and standardised for nutrition surveys [35, 42]. Since 2006 the adoption of the SMART guidelines for nutrition surveys has greatly enhanced the reliability of findings due to the rigorous emphasis on data quality [40, 43]. Key aspects are first, standardized automatic checking (so-called “plausibility check”) of the quality of anthropometric data [44] and second, substantial improvements in sampling techniques at the last stage of sampling, with an emphasis on simple and systematic random selection, and discouragement of the EPI “spin the pen” selection method originally promoted in 30 × 7 and 30 × 30 designs [41, p. 30]. The SMART guidance advocates an almost identical approach to the 30 × 30 cluster sample, apart from the calculation of sample sizes and the number of clusters required. In most cases these lead to a much smaller sample size of between 400 and 600 subjects, compared with 900 for a 30 × 30 design, resulting in substantial savings in cost and effort.

Given the high cost of good-quality surveys, attention recently has been given to develop nutrition survey methods that require relatively small samples without a great loss of precision. Various sampling designs have been proposed and applied including designs with cluster sizes similar to those of vaccination coverage surveys (33 × 6) as well as 67 × 3 [45, 46]. While these designs offer advantages of statistical efficiency, this is less of a benefit for outcomes in which most variability occurs within clusters, such as wasting, compared with outcomes such as vaccination coverage for which a higher proportion of variability occurs between clusters. Also, although the smaller sample size in these designs might be expected save cost and time, these benefits may be lost for a design of 67 × 3 (clusters × children) because of the cost and time needed to travel between clusters and properly sample children in each village [46].

As surveys have become less expensive during the last decade and their findings more trusted it has become more common to use cross-sectional surveys to monitor the nutrition situation. For example, the agency Action Contre la Faim (ACF), which is active in areas where there are no formal nutrition surveillance systems but which regularly implements cross-sectional surveys, has developed expertise in using data from these surveys to detect and interpret trends, for example in South Sudan [47]. Recently a lower-cost rapid survey approach has been developed for use in emergency contexts, adapted from the traditional SMART method, called Rapid SMART [48]. By stipulating a fixed sample size of 25 × 8–12 (clusters × children) and collecting data on small number of indicators, the surveys can achieve sufficient precision as well as disseminate key findings within 1 or 2 days of data collection [49]. Evidence from applying this approach in South Sudan indicates it can be feasible to obtain regular representative nutrition data in very challenging contexts, notwithstanding difficulties related to inaccurate population data, poor access and logistics, and a lack of technical capacity [49].

The advantages and disadvantages of using repeated sub-national cross-sectional surveys for surveillance were summarised in the previous review [14]. With respect to institutional management, generally non-governmental organisations (NGOs) undertake the surveys in collaboration with national governments, and may be funded by development or UN agencies. Examples are: small-scale SMART surveys in Garissa and Mandera counties of Kenya [50]; in the Karamoja Region of Uganda [51]; in Upper Nile State, Malakal, South Sudan [52]; and full SMART surveys in South Sudan [53]. Other organisational models exist, for example in Somalia survey data are collected by local NGOs but the process is coordinated by a UN body because of the security situation [54]; in Bangladesh the data are collected by an academic institution in collaboration with an NGO and the government [55]; and in Nicaragua, government agencies collect data [56].

#### Primary data collection: community-based sentinel sites

As is the case for repeated cross-sectional surveys, nutrition assessment in community-based sentinel sites is an approach most often used by NGOs. Data are collected periodically in communities selected because they are in an area that is highly vulnerable to malnutrition or that is typical of a livelihood zone or area. Typically 12–50 children are studied per site and data are collected every 1–3 months. Children are sampled randomly within the sites but the sites themselves can be sampled either purposively or randomly within the district, livelihood zone or ecological zone that has been sampled purposively. A new sample of children can be selected each time at each site, as in South Sudan [57] and Zimbabwe between November 2004 and October 2006 [58]. Alternatively, the same children can be studied repeatedly, with replacements when children become older than a threshold age, are lost to follow-up, or die, as in the Listening Posts Monitoring System [59] implemented in Zimbabwe by Save the Children and in Burkina Faso by ACF.

The advantages and disadvantages of this general approach to nutrition surveillance were described in the previous review [14]. In summary, the advantages of this approach over traditional surveys are that it is quicker and costs less; that fewer sites are included so more detail on causes can be collected; and that community members can become involved in the data collection, leading to assessments which are more participatory. The main disadvantage is the unknown level of bias which is likely to vary on a case-by-case basis depending on the environmental context and exact methods applied. With respect to bias, further evidence has since been published of how, during the process of surveillance, the nutritional situation in the selected sites may become progressively different from the rest of community that they were chosen to represent [60, 61]. This is due to the inputs of the survey teams who may provide education, advice and counselling; treating illness or referring malnourished children to a treatment programme; and providing employment and spending money within the community [62]. In addition, the nutrition situation might improve simply because children are being measured repeatedly, since parents might increase their efforts to feed and care for their children as they know that they are being observed and evaluated. This phenomenon is referred to as the “Hawthorne Effect” [63], and can affect the validity of evaluation findings or observed trends in nutrition status [64]. Also significant movements of populations over time either in or out of communities may invalidate the representativeness of the sentinel sites [65].

#### Primary data collection: schools

The approach of collecting data about school children differs from other methods of primary data collection for nutrition surveillance in that there is generally no sampling: all children at a particular stage or stages of schooling are included. Data collected on school children was used for nutrition surveillance in Central America in the 1980s [9, 66–68]. In some countries periodic censuses of the height of children enrolled in the first grade of primary schools are still undertaken, for example, in Nicaragua [69]. In Guatemala there were height censuses of schoolchildren in 1986, 2001 and 2008 and the data were used to monitor stunting rates and target local interventions [70]. In the Seychelles there was a school-based surveillance programme between 1998 and 2004 that included all pupils in kindergarten and in the 4th, 7th and 10th grades, giving an age range from 5 to 15 years [71]. In Palestine, the system is slightly different in that only a sub-sample of sentinel schools are included, then data are collected each year on the height, weight and food habits of all children in the 1st, 7th and 10th grades of those schools [72].

This approach costs less than traditional surveys, while the disadvantages relate to the potential sources of bias. For example, if the proportion of children entering the first grade of school within a few years of the official entry age is lower than about 80 % then the children registered at school are not likely to be representative of those the general population [70]. Also bias is introduced if the dataset is not complete and if missing children do not occur at random, for example if attendance at school is low and it is the children of low socio-economic status who are most likely to be absent.

### Collection of secondary data for nutrition surveillance

For surveillance purposes, the advantages of using data from health systems are generally that the costs of undertaking primary data collection are avoided; they are available more quickly than survey data; and they have a greater breadth of coverage. The disadvantages are that the data are rarely complete; the data are usually not representative, as the poorest and more vulnerable sections of the population are less likely to attend health facilities than other people because of limited access and cost of health services [73]; and data are often of poor quality due to factors including poor motivation, lack of supervision, inadequate feed-back, and overburdening of staff by multiple reporting requests [74]. Also, as discussed above, nutrition-related data from health-facilities cannot necessarily be considered as “…a by-product of patient care…” [74, p. 26] unlike data relating to coverage, utilisation and management of services, and to morbidity and mortality. Anthropometric measurements may not be made “routinely” unless the government requires the reporting of anthropometric indicators.

#### Secondary data: admissions to feeding centres and to community-based management of acute malnutrition (CMAM)

Examples of countries in which data from feeding centres or CMAM programmes are used for nutrition surveillance are Ethiopia [28], Sudan [57] and Afghanistan [75]. Also since 2006, Niger has included data from feeding centres in its food security early warning system [76]. A comprehensive monitoring and reporting package for all CMAM components has been developed.^1^ While several countries have compulsory national reporting systems for CMAM data, the separate national systems often do not fully align with the new reporting package, leading to parallel reporting systems and sometimes differences in calculating performance indicators. The use of the recommended guidelines and CMAM reporting software [77] could promote the consistent reporting of categories and indicators, and thus enable data on performance to be compared between clinics, implementers and countries.

New survey methods have been developed to estimate the coverage of routine CMAM programmes from district to national levels including Centric Systematic Area Sampling (CSAS), Semi-Quantitative Evaluation of Access and Coverage (SQUEAC), Simplified Lot Quality Assurance Sampling Evaluation of Access and Coverage (SLEAC), and Simple Spatial Survey Methods (S3M) [78]. The implementation of these methods has enabled coverage audits to be done more regularly and quickly.^2^ Thus, data on the coverage of CMAM services and on the prevalence of wasted children is becoming more widely available [79] and will play an increasingly important role in nutrition surveillance. The advantages and disadvantages of this approach to nutrition surveillance, including sources of bias, were described in the previous review [14].

#### Secondary data: anthropometry data from clinics

The main source of anthropometry data collected routinely through health systems comes from growth monitoring of children in clinics from which data are compiled and used for surveillance. Some surveillance systems are or have been based on such data alone, for example the Botswana National Nutrition Surveillance System has done this since 1978 [80, 81]. Birthweight can also be used for surveillance, as in Nicaragua [69, p. 24].

Clinic-based growth monitoring data may provide early warning of a deteriorating health and food security situation, as in Ghana between 1981 and 1983 [82, p. 7]. Such data may aid decisions about targeting interventions by identifying vulnerable geographic areas, and the data may be especially useful in emergencies where there is physical insecurity and it is not possible to carry out surveys, such as in parts of Somalia.

The sources of bias for growth monitoring data in addition to the general biases mentioned above for data collected in health facilities, were described in the previous review [14]. Several papers have documented the discrepancy between estimates of child malnutrition derived from clinic-based nutrition surveillance and those derived from national surveys [81, 83–86].

Sentinel clinics can be chosen at sites because they are typical of a livelihood zone, or because the communities are vulnerable, and then resources are directed at these key clinics to strengthen the processes of data collection and analysis. For example ACF collected data from specific clinics in Somalia and ensured that they received extra resources to ensure a high quality growth monitoring programme [82, p. 29]. In Mozambique, data are collected at one clinic per district, all located in district capitals. This selection was based on practical considerations, since compared with more remote clinics, district capital health clinics are better staffed and equipped and therefore have a better chance of being able to sustain the surveillance system [25]. In Malawi, five clinics were selected within each district, and in addition to anthropometric data, data on food security, morbidity and water and sanitation were collected each month from 10 of the 70 households sampled once a year from each clinic [87]. In Karamoja, Uganda, 15 new centres were established where there were gaps in data sources [88].

#### Secondary data: anthropometry data collected in the community

There are several ways that anthropometry data are collected through health systems in the community including: community-based growth monitoring, such as in Bangladesh [82, p. 9]; screening children for referral to feeding programmes, such as in Chad [89], in Maharashtra in India [90], and in Haiti [91]; and screening children for malnutrition incorporated into Child Health Days as one of a package of activities, such as in some districts of Ethiopia in the Extended Outreach System [92].

The advantages and disadvantages of using these data for nutrition surveillance were described in the previous review [14]. The further advantages are that if coverage is good and selection bias low the data can provide useful information on trends in prevalence of acute malnutrition and, when disaggregated by gender, age and geographical area, the data can provide useful information on which groups are most vulnerable.

### Combination of methods

Surveys that provide representative data and reliable estimates are expensive, and repeated surveys more so. Consequently, one approach to surveillance involves undertaking surveys only in areas that have been identified as experiencing deterioration in nutritional status or food security by another method, such as monitoring admissions to feeding programmes or data from community sentinel sites. This approach was proposed by Pelletier and Msukwa [93] who recognised that data being collected for surveillance mainly from growth monitoring and national sample surveys were not sufficient for certain types of planning decisions. They suggested that future surveillance systems should build local capacity to undertake ad hoc investigations, which would support analyses based on existing data. This is the current approach to surveillance in Ethiopia [28, 94] and it replaced the formal surveillance system run by Save the Children which was based on annual cross-sectional cluster surveys with quarterly longitudinal follow-up of children [95]. When using a combination of methods for surveillance, the prevalence rates derived from the ad hoc surveys are representative only of children in the badly affected areas and cannot be generalised.

### Considerations when choosing a strategy and methods for data collection

The approaches applied to nutrition surveillance were outlined above. This section discusses the application of these approaches for surveillance, taking context and objectives into account. The main issues are related either to the design, meaning the general strategy adopted, or to the methods, meaning the specific data collected and the choice of techniques for doing so.

The ultimate use of the information is what determines the optimum strategy and methods for surveillance. For example, to monitor progress towards targets, the need is for accurate and precise estimates of prevalence, while for timely warning of a nutritional problem the ability to detect a trend is more important than the accuracy of the absolute values. At the end of this section, the best combinations of strategy and methods are described for what are arguably the three most common objectives of surveillance, bearing in mind that surveillance systems often aim to serve more than one purpose.

#### Design: primary versus secondary data collection

It is unfortunately the case that in many countries clinics are perceived as a mechanism through which nutrition data can be regularly accumulated, with apparently no understanding of the effect on other activities and the lack of utility of the resulting data. Mandatory reporting of nutrition indicators, including stunting and wasting, pose a huge burden on already overstretched health workers, and sufficient provision of the training, supervision and equipment to enable good quality data to be collected is not feasible in low-income contexts. Also, as described above, there is considerable evidence that data obtained from children brought for health services are not representative of the general population. These considerations mean that data from weighing programmes, such as from children under 5 years of age attending clinics, or from new-borns at attended deliveries, must be treated with caution and not used to judge the severity of a situation.

As discussed below in the section on quality control, interpretation of secondary data must take into account possible measurement error, as well as potential changes in bias in case selection which may affect comparability of data over time. If the likelihood of change in bias is judged to be low, data from health systems can provide useful indications of local trends in nutrition status, and of national trends during the periods between large-scale surveys such as DHS or MICS [29]. Of course it is preferable to base decision-making on representative data, and this can only be obtained from surveys. The feasibility of conducting annual national nutrition surveys has recently been demonstrated in West and Central Africa [96, p. 13]. These provide representative data at the level of first administrative divisions within countries, and through rigorous attention to budget details, the costs of surveying each such stratum is normally about $10,000 (personal communication, Robert Johnston). This model of surveillance is preferable to dependence on health-systems data as it is not expensive and poses no burden on routine health systems.

#### Design: longitudinal versus repeated cross-sectional data collection

By repeatedly including the same children in consecutive rounds of data collection, there is less random variation in measurements compared with sampling new subjects at each round. Consequently the statistical power to detect a significant change is greater. The disadvantage of such longitudinal data collection is that it is labour intensive and expensive to repeatedly find and measure the same individuals, something that is usually done only in cohort studies in wealthy countries. Children can be lost to follow-up, as in the system using data from sentinel clinics in Malawi [97, 98], and there may be bias introduced by the Hawthorne effect of repeatedly studying the same children, as noted in Northern Nigeria [60]. Furthermore, if children are lost to follow-up and are replaced, the sample becomes a mixture of longitudinal and cross-sectional samples, which makes the analysis more statistically complex and there may not be enough statistical power to detect changes. The inclusion of new children in the sample also changes the mean age of the subjects between rounds, which may be a problem since values of anthropometric indices change considerably during the first 2 years of life compared with the next 3 years. For example in the Zimbabwe Listening Posts system, the age structure of the sample changed from one round to the other, making it very difficult to interpret mean weight change and mean mid upper arm circumference [99].

#### Design: repeated sampling of sites versus fixed locations

The choice of sampling methods depends on how the information will be used. If accurate estimates are required, for example to obtain prevalence rates at national or district level or for ecological zones, then the data must be statistically representative of the population in those levels or zones, so probability sampling is needed. For such surveys, a clear definition of the target population to which the results can be generalized is needed, as well as careful attention to the sample size, close supervision of interviewers and assurance of a high response rate [32]. Large changes in the population due to mortality or migration may bias findings between repeated surveys.

To reduce the cost or for the sake of convenience, smaller samples are possible by choosing individual villages or clinics that are thought to be typical of larger areas such as a livelihood or ecological zone or in an urban area, and designating them as sentinel sites which are repeatedly monitored. Purposive sampling can be used to select health facilities or villages as sentinel sites based on their features in relation to the indicators being monitored [31], or random sampling can be used to select sites within the livelihood or ecological zone. Even if random sampling is used, the credibility of the findings is more open to question compared with surveys using probability sampling because of the purposive selection, the small size of the sentinel sample and because of the evidence that the true situation can be masked over time due to the attention paid to the site by the survey teams. Therefore to reduce this last form of bias and provide the most reliable data, it is best to sample a new site or sites within the ecological zone at each round, or at a minimum, each year.

If probability sampling is used to select the children to be included in each round of data collection, sentinel sites can potentially provide data that is representative in the statistical sense of providing an unbiased estimate of what the population of the zone is like. However, it is not possible to know whether this is true in individual situations without also undertaking good quality anthropometric surveys of children in the ecological zone using probability sampling, as such surveys are accepted to provide data that are representative of the population. These data could be used as a ‘gold standard’ against which the sentinel site data could be compared.^3^ Such research to validate the sentinel approach would be helpful, as would guidance on how to minimise bias. Yet even if such research had been done, the guidance had been drawn up, and the methods then applied, the small sample sizes involved would mean uncertainty would still exist over the reliability of findings from sentinel sites in individual situations.

#### Methods: sample size

Surveys need to be designed to ensure that there is sufficient statistical power to detect significant differences between rounds of data collection and at least three data points are needed to detect a trend. When calculating the sample size needed to detect a difference in prevalence rate between two rounds, it is necessary to estimate the prevalence rate at the first round, and the difference in prevalence between rounds that would signify a meaningful change [100]. In addition, if the survey uses multi-stage sampling, such as cluster sampling, then the design effect, calculated from the ratio of the variances within groups and between groups, must be used to adjust the estimate of required sample size.^4^ If no design effect for the prevalence of wasting can be calculated yet, then it is typical to apply a value of 1.5 [42]. If estimates are to be made using disaggregated data, for example by sex, geographical area or socio-economic status, this analysis must also be taken into account during the sample size calculation, as a larger sample will be needed to have the same statistical power to detect differences between surveys in estimates for the sub-groups.

#### Methods: frequency of data collection

The frequency of data collection depends on which indicators have been chosen and these, in turn, depend on the objectives of the surveillance. For timely warning and programme planning in emergencies the prevalence of wasting and the prevalence of a small MUAC in children <5 years are good indicators of acute malnutrition as they can increase quickly during a crisis due to food insecurity or during outbreaks of disease. In contrast it takes many months or years for a change in the prevalence of stunting to be detected, so stunting is a good indicator of the food, health and care environment over the long term that contributes to chronic malnutrition and is therefore a particularly useful indicator to track the process of development locally and at a national level. Similarly, some indicators of exposure may need to be collected less frequently than others as they change little over time. For example, food production is seasonally influenced, so agricultural data should be collected frequently to capture any variation, while indicators of hygiene such as the prevalence of latrines, or of feeding practices such as the duration of exclusive breastfeeding, are likely to change slowly.

Seasonality is an important source of variability in anthropometric data: if the surveys are to be annual, they should be undertaken at the same time each year so that long-term trends in data over time can be distinguished from seasonal variation. In areas with vulnerable populations, a case may be made for collecting data more frequently than annually, and the timing of the intervening surveys should be chosen to correspond with peaks and troughs in nutritional status due to seasonal variation. However, if the seasonal variation has a very short periodicity, the value of collecting data frequently to capture all the short-term variation may not justify the additional effort and cost. For this reason the frequency with which data were collected in Bangladesh was reduced from six times each year in the Nutrition Surveillance Project [101] to three times a year in the FSNSP [55] as the objectives of the system changed from disaster preparedness to become a tool for policy and programme planning. Given that there has been a surveillance system in Bangladesh since 1990 and therefore a wealth of historical seasonal data exists, perhaps even this frequency of data collection is no longer necessary except in the most vulnerable areas of the country. In the Listening Posts project in Zimbabwe which had the objective of detecting shocks and their nutritional effects at local level, market price data were collected every month and nutrition data every 3 months [102].^5^

#### Methods: type of data to be collected

As described at the start of this paper, in principal nutrition surveillance involves the regular and systematic collection of data relating to nutritional exposures as well as outcomes since, for decision-making, data are needed on the likely causes of poor nutrition. However, it is only the first category of approaches described above, those relating to primary data collection, in which additional variables can be collected for children on which they are reporting. It is generally not feasible to collect additional exposure variables through health systems, although the Malawi Integrated Nutrition and Food Security Surveillance (INFSS) system is an example of a clinic-based system at which food security data were collected each month from one in every seven households with children from whom anthropometric data were collected [87]. Also, it is important that the number of additional variables collected is kept to a minimum, to reduce risk of overload and risk of poor quality of data.

In the UNICEF conceptual framework of malnutrition [103, 104] the underlying causes of undernutrition—food insecurity, health services and care practices—are distinguished from the basic causes of undernutrition, which include environmental, economic and socio-political factors. The framework can be used to help identify the indicators to be recorded in a surveillance system and to try to assess the factors that drive malnutrition.

Indicators of immediate factors are most often measured for individuals and relate to their health and diet; for example, the illness of a child in the past 2 weeks and breast feeding practices. Underlying factors are most often measured at household, community or higher levels and relate to household circumstances, the economic environment and the climate, for example water and sanitation facilities, household coping strategies, and market prices of food. There is likely to be a time lag between changes in the values of immediate indicators and changes in the values of the nutritional outcome variables, and a longer time lag for underlying factors. Data on variables of immediate and underlying causes of malnutrition may be associated with anthropometric status, but on a cross-sectional basis, correlations with nutritional outcomes will be weaker with underlying causes than with immediate causal indicators. Cross-sectional data cannot be used to determine causal relationships. However, if data are collected systematically and periodically, then the resultant data series can enable stronger causal inferences. For example, a change in an immediate cause might be related to a subsequent increase in the prevalence of an outcome. The time lag to detect a significant effect will be longer for variables related to height than to weight as height changes proportionately more slowly than weight, and height cannot be lost whereas weight can.

Indicators of processes, such as the delivery of vitamin A capsules to children or the number of children treated for severe acute malnutrition at health facilities, also help to assess the quality and coverage of health services and provide information about context.

Food insecurity is a key underlying cause of malnutrition, so indicators of food security are always of relevance in nutrition surveillance. However some surveillance systems explicitly monitor food security as an outcome, such as the Food Security and Nutrition Surveillance Project (FSNSP) in Bangladesh [55] and the Integrated Nutrition and Food Security Surveillance (INFSS) System in Malawi [87]. The assessment of food security adds significantly to the resources needed for data collection and analysis, with respect to materials, expertise, training, software and time.

There are four types of food security indicators that can be included in surveillance systems: energy deprivation, monetary poverty, dietary diversity, and subjective, experiential indicators such as the Household Food Insecurity Access Scale [105]. Some stakeholders in surveillance may resist accepting findings based on subjective indicators. Also, confusion and scepticism can be caused by the inconsistent way in which some of these indicators are calculated. Different indicators of dietary diversity for young children, women of reproductive age and households each use a different number of food groups: seven for young children, ten for women and 12 for households [106–108]. The consumption of at least four of seven food groups defines a “minimum diversity” score for assessing the nutritional quality of young children’s diets [107], and consumption of at least five of ten group food groups defines the indicator to assess the micronutrient adequacy of women’s diets [108], however cut-off points in terms of number of food groups to indicate an adequate or inadequate dietary diversity have not been endorsed for the household dietary diversity score [106].

In order to compare the extent to which different countries are affected by common factors such as global climate change and food price shocks and to monitor progress towards targets, there is a need to harmonise the use of household survey-based food security indicators [109]. In contrast, for timely warning of a nutritional problem at a sub-national level, data relating to local livelihoods are necessary [110] and context-specific indicators are most appropriate, ideally chosen using participatory approaches [111].

#### Methods: data quality

The collection of original data for population nutrition surveillance will always involve errors, they are impossible to eliminate completely. There are two types of error, systematic and random. For nutrition assessment, a systematic error, or bias, will affect the mean of the normal distribution of Z scores, while random errors will not influence the mean or median, but will increase the variance. Both types of error influence the estimates of the prevalence of malnutrition—for systematic errors the prevalence will be under- or over-estimated depending on the direction of the bias, while for random errors the prevalence will always be overestimated because the tails of the distribution become fatter due to the increased variance [38].^6^

Random errors are due to chance, and lead to a deviation of the estimated value of a variable obtained from the sample from the true but unknown population value. Random errors occur mainly for two reasons: sampling error, the error caused by including data from a sample instead of the whole population, and measurement error, the difference between the value recorded and the true value for each subject caused by variations in the measuring process. Sampling errors cannot be prevented, but if the sample size is sufficient,^7^ and random measurement error is minimal, the impact of random error on the effectiveness of surveillance will be small. However large amounts of random error in anthropometry measurements can lead to a dramatic overestimate of the prevalence of undernutrition. Therefore it is critical to minimise random error in measurement by using standardized techniques and rigorous quality-control procedures as described below. Unfortunately in anthropometric surveys, attempts to reduce sampling error by increasing the sample size can be counter-productive because the requirements for training and supervision increase, as do surveyors’ fatigue which may affect their accuracy.

Systematic errors lead to a deviation from the correct result due to consistent faults or mistakes in sampling, measurement or recording. For example if clothing is not removed from a child the resulting positive bias in the measurement of weight will lead to an underestimation of the prevalence of wasting.

There are several ways to limit errors and thus to maximise the potential value of the data collected for surveillance. First, specialist, well-trained staff is essential for data collection. Ideally staff should be trained or re-trained before each round of data collection. As well as optimising data quality, there are cost-savings related to having rounds which are sufficiently frequent, or which include different areas in turn, to enable teams to be employed continuously and to avoid the need to recruit and train new staff for each survey. Ideally, data collectors are locally recruited or the same teams return to the same communities, as this promotes an in-depth understanding. Second, quality control procedures can be applied such as random spot-checks on staff to verify that data have been collected and are correct. For example, between 1990 and 2007 the Bangladesh Nutrition Surveillance Project used staff of well-established local NGOs as field teams to collect data and the lead agency, Helen Keller International, sent quality control teams to make random spot checks on data collected the previous day by staff [101].

Hand-held electronic devices to record data have the potential to improve the quality of nutrition data by capturing raw data rather than aggregated counts, and by adding automatic quality checks to software to highlight outliers or prevent missing data. The latter function provides both instant quality feedback in the field, as well as summary judgment on whether the data conform overall to minimum quality standards and are therefore fit for use in decision-making. Although no rigorous evidence exists of improved data quality related to the use of mobile technology, a relevant evaluation in Indonesia is underway [112]. The use of hand-held devices eliminates the errors introduced when data are transferred from paper to computer, together with other problems such as legibility or damage to forms caused by transportation or humidity. During growth monitoring, nutritional status is often determined by manually plotting measurements on a growth chart, so errors and imprecisions are more likely than with an electronic calculation. The use of mobile technology for improved growth monitoring has been described from India [113], Sri Lanka [114] and Malawi [115], and there are ongoing projects in Madagascar and Mozambique [116] among others.

#### Objectives: long-term monitoring for national and local policy and planning

In the past, national surveillance systems were mainly based on data from child growth monitoring. While such data are not ideal, the information provided useful guidance to formulate general national nutrition policies and plans [13]. More recently, valuable nationally representative data have been collected every few years by the DHS and MICS, which enable long-term trends to be detected and to monitor progress towards international targets such as the MDGs and WHA global targets. However for detailed national policy and planning purposes these surveys are not frequent enough while the data cannot be disaggregated sufficiently to identify locations or socio-economic groups most in need of interventions.

In order to monitor national trends better, it is feasible in all but the most unstable contexts to collect nationally representative nutrition data at a greater frequency than is currently provided by the DHS and MICS. There is a model for this in Nicaragua [56] where nationally representative data are collected annually, and regionally representative data are collected every 3 years. As discussed above, the system of annual nutrition surveys implemented in West and Central Africa [96] demonstrates that this model is feasible. Over-sampling of vulnerable areas or at the request of a donor for evaluation purposes, such as in the Bangladesh FSNSP, can provide detailed annual data for use at regional and district levels.

With respect to the level to which data are disaggregated for local planning purposes, repeated surveys at an informative level of disaggregation would be expensive and are not necessarily the best approach, instead there is potential to better use data from health systems for local monitoring [29]. For example, data from clinic and community growth monitoring and mass screening programmes can be compiled as part of normal operational processes and sent to a higher administrative level. This can provide regular estimates of the prevalence of child malnutrition in districts as well as data on the implementation of programmes. It is important that the raw data as well as the aggregate numbers are reported, so that the quality of the anthropometric measurements can be checked.

Given the high cost of undertaking surveys we suggest that the most pragmatic strategy is to use surveys conducted using probability sampling to estimate prevalence rates, and use sentinel sites and health-systems data both to indicate trends between large-scale surveys and to identify which locations need surveys using probability sampling. This requires that the data reported from sentinel sites and by health facilities are collected using consistent methods so that any increase in prevalence is indicative of a change in circumstances, not a change in method. This may require supportive contextual data, for example reports of an outbreak of disease, a crop failure or an influx of refugees, which could warrant a survey to assess a deteriorating situation. An example of this approach can be found in Kenya where health facilities supported by the agency Concern are recording the number of cases per month of children found to be acutely malnourished plus local contextual data to detect a surge in need for treatment and trigger an automatic response by local health authorities [117].

Such programmes are not feasible in every country. An alternative option is to have a mix of so-called ‘thick and thin’ rounds of primary data collection where the ‘thick’ rounds are conducted less frequently but collect data on the widest range of variables, and the ‘thin’ rounds collect data on fewer variables, especially those that may change more rapidly [24]. Another option is to have a mix of simultaneous ‘thick’ and ‘thin’ data collection, whereby detailed data are collected only at some specific sites each round and in the remaining sites, data on fewer variables are collected.

As described above, seasonality is an important source of variability in anthropometric data, so the timing of data collection must be carefully planned so that tracking of trends in data over time is not hindered by seasonal variation.

A range of nutritional indicators is needed to fully characterise the nutrition situation. Recent evidence does not support the current degree of separation of wasting and stunting into simple acute and chronic conditions [118], and so for policy and programming purposes it is important to collect data on both indicators as a minimum. It is also important to disaggregate findings by age and sex, and ideally to include adult women as well as young children, because adult members of a household may protect children from the harmful effects of shocks by sacrificing their food intake, as was observed in Bosnia in 1993/1994 [119] and during Indonesia’s drought and financial crisis in 1997/1998 [120].

#### Objectives: evaluation of the nutritional impact of programmes and projects

Well-designed and implemented randomized controlled trials (RCTs) are considered the “gold standard” for evaluating an intervention’s effectiveness, because the processes used during the conduct of an RCT minimise the risk that confounding factors will influence the results [121]. Since evaluations of nutrition programmes cannot easily involve randomised controlled trials unless they apply a stepped wedge design [122], they typically aim to give plausible estimates of effectiveness rather than probabilistic conclusions [123]. Evaluation studies with plausibility designs are usually sufficient for decisions about whether to continue, modify or replicate public health programmes [124]. For such impact evaluations either a “counterfactual” must be identified to assess what would have happened to similar individuals in the absence of the intervention [125], or there must be considerable variation in exposure to the intervention to detect a dose–response relationship. The choice of indicators must relate to the objectives of the programme, and substantial resources are required for both data collection and analysis of evaluations [29]. Outcome data ideally should be disaggregated by age groups and sex to check for differences, which has implications for the required sample size.

The evaluation of programmes has long been identified as one of the specific objectives of nutrition surveillance [2]. Since most early systems used secondary data, it was not realistic to think that such data alone could be used for this purpose, notwithstanding that administrative data can be a useful adjunct to original data collection to evaluate programme effectiveness [126]. In order plausibly to attribute changes in nutrition outcomes to programme activities, this requires a prospective design with some form of both before and after measures, and some comparisons of subjects with and without the interventions (thus a counterfactual), or with significant variation in exposure to the intervention [29]. Thus even current surveillance systems that collect primary data cannot be used to evaluate the effectiveness of large-scale programmes unless additional information is collected. For example, in Bangladesh, the FSNSP data collection has been expanded to collect data in zones included in the programmes of the US government’s global hunger and food security initiative ‘Feed the Future’.

In contrast to estimates of effectiveness, it is relatively straightforward to monitor the implementation of programmes and delivery of services or projects as long as data are collected on process indicators such as access to and use of services, as occurs in the system in Nicaragua [56]. In most countries, some process indicators are collected in any case as part of large nationally representative surveys including DHS and MICS. In this way it is possible to check if services are being delivered to the target group.

#### Objectives: timely warning

There are many examples of nutrition surveillance systems in which data have been used for early warning of a deteriorating nutrition situation to enable mitigating actions to be taken. For some systems this is the primary focus, such as the Nutrition Surveillance Programme in Ethiopia between 1986 and 2001 [95], while for others timely warning is one of a number of goals. For a warning to be timely the rapid collection, analysis and reporting of data on a few predictive indicators is needed. New technology to collect and transmit data could be really helpful in this regard.

Due to the time lag between exposure to causes of malnutrition and a change in nutritional outcomes, indicators of anthropometric status are generally not useful for the purpose of prediction. Instead data that can be used to predict potentially harmful trends in food security or disease are more helpful, such as data on food prices, rainfall and outbreaks of disease. But as long as the data are reported quickly, anthropometry data can be valuable for purposes other than prediction, such as monitoring nutritional outcomes, assessing intervention delivery and coverage, and for modifying targeting based on demographic groups or geography.

In some contexts, changes in food intake and nutritional status may occur at an early stage of a food crisis when a population’s coping strategies are not yet damaging or dangerous, for example when meal frequency or the quantity or quality of the diet is reduced. As such changes may also occur as a normal response to seasonal shortages of food, abnormal responses can only be distinguished when compared with data from previous years [95], so data from surveillance can be valuable for prediction. For example in Indonesia, surveillance detected that the quality of the diet, not the quantity, had changed during an economic crisis when reduced access to animal and fortified foods led to lower dietary intakes of iron [127].

Some anthropometric measurements or indices can change relatively rapidly, such as MUAC or weight-for-height, because body weight can be lost as well as not gained. Height-for-age changes less rapidly, so is a less sensitive indicator of short term changes in diet, disease or caring practices. For timely warning in most contexts, the collection of data on MUAC and weight-for-height would ideally occur every 2–4 months. Findings from the INFSS system in Malawi where data were collected monthly [87] indicate that, except in very exceptional circumstances, changes in prevalence rates are not sufficient to justify the extra work, costs and potentially lower quality of data associated with monthly data collection.

As discussed above, sentinel systems can be useful for detecting trends even though the data may not be statistically representative of the population. Such data can be particularly useful to provide a timely warning in situations in which there are security risks when conducting nutrition surveys, such as in parts of Somalia. Similarly, while health systems data are recognised as being biased, they can be useful for timely warning in places where access or resources are limited. For example, a downward trend in anthropometric status derived from growth monitoring data indicated that an emergency was developing in Ghana in 1983 [82], and feeding programme data identified a surge in demand for services in Niger during a crisis in 2005 [128].

### Collation, analysis, and interpretation of data

Before any data can be considered, its quality needs to be assessed, whatever its source. The most common errors occur during measurement, when recording data or during data entry, especially when using paper forms to collect data. As a part of the analysis of data collected during SMART surveys, indicators, scores and statistics of data quality are calculated by ENA software [43] and for some values the probability that they could have occurred by chance is estimated. Ranges are then used to describe the overall quality of the survey and arbitrary cut-offs are used to decide whether the data are acceptable or not [44]. But such an analysis is usually retrospective, so while the checks inform on data quality it is too late to act upon the information. It is therefore not a substitute for accurate equipment and good staff training before the survey begins.

These sorts of checks are rarely done for secondary data. There is great interest in using hand-held devices to lead health workers through the process of assessing and treating children. As described above, the use of hand-held devices to record data in the field is allowing measurement and data recording errors to be reduced [113] and the effect on data quality is being evaluated [112]. Critical functions of mobile technology are to capture raw data rather than aggregated counts only, and to perform automated quality checks on these raw data. These checks enable both instant feedback on quality in the field, and also judgment as to whether the data meet minimum quality standards, and can therefore be used for making decisions. However, while substantial measurement and data entry errors can be flagged, checked and corrected if values are outside an expected range and are due to human error rather than to inaccurate equipment, errors in reporting a child’s correct age are a persistent problem, particularly if age is estimated from stature, so the prevalence of age-related indicators may be underestimated.

When designing any surveillance activity, it is important to consider issues of quality ascertainment and design processes of monitoring and routine quality checks, whether on survey data (as is currently done in SMART [43]), data from community sentinel sites, or health systems data. Procedures for ascertaining data quality must be defined during the design stage, and guidance drawn up for interpreting findings from such analysis, to help users judge the reliability of data. While guidance exists for assessing quality of survey data [44] there is no guidance in the literature for quality assessment of data obtained from other methods of data collection. Given that secondary data are useful to ascertain trends rather than prevalence rates, the guidance for surveys needs to be modified, since the relative importance of the various quality criteria is different for secondary data compared to primary.^8^

Once the quality of data has been assessed, the people analysing surveillance data need to make decisions about how best to undertake the analysis and present the findings in order to maximise the likelihood of response, if one is needed. Some issues they need to grapple with include: deciding whether to compute and present the mean values of indices or the prevalence of indicators, ideally both; accounting for the stratification or clustering of subjects when calculating confidence intervals; determining the level to which data can be disaggregated; separating periodic variability from secular trends; and choosing reference values to do calculations. Analysts also have the important role of interpreting the data and identifying the practical implications of the findings in order to highlight them to the users of the information. This is not straightforward: for example, a similar prevalence of acute malnutrition may differ in significance depending on the context. Also, unless the analysts understand what the underlying causes of nutritional disorders are likely to be, the appropriate response may not be identified.

Then there is an essential step which is often missing in surveillance systems: expressing findings in language that is comprehensible to others, for example to people in civil society, managers in the UN and donor agencies—people who are not nutritionists but who need to understand what is going on. For surveillance systems to be effective “… information should be functionally disaggregated so as to guide decision-makers, rather than being simply undifferentiated data for technocrats” [129]. The overall impression gained from many reports of surveillance findings is that insufficient thought has been given to the needs of busy decision-makers who rarely have time to read long documents, and are especially unlikely to do so if the contents are complex and presented in small print. There is much potential to improve communication, for example by producing separate reports for different types of users, and having a stronger focus on the policy and programming implications of findings than on the data alone.

It is clearly important that the information derived from surveillance activities gets to those with the potential to take decisions that lead to action, so it is crucial that the outputs of surveillance are disseminated effectively. We are not aware of instances of information from national surveillance systems being disseminated from central level to a level lower than the district, so many health workers remain uninformed. Methods used to distribute information include workshops and presentations for stakeholders; bulletins and policy papers distributed as hard copies, on a CD-ROM or over the internet; papers published in academic journals; and even books. Cleaned data from every second year of surveillance were also distributed on a CD-ROM for the Indonesian Nutrition Surveillance System [130] and Bangladesh NSP [131]. The DHS Program is authorized to distribute survey data files for further analysis at no cost to the user provided that the user registers first.

Ideally when a nutrition surveillance system is designed, the potential decision-makers are involved with developing an analytical framework for how the information will be used. This strengthens the system’s credibility and so increases the likelihood of a response where necessary [132].

### Harnessing developments in electronic technology

Taking advantage of technological innovation has been a theme through the history of nutrition surveillance, from drawing on food security assessments informed by satellite data in the 1980s, computer assisted interviewing in the 1990s, and the use of mobile phones and development of “real time monitoring” (RTM) in the last decade in which “real time” means the most recently collected data.

**Fig. 2:**
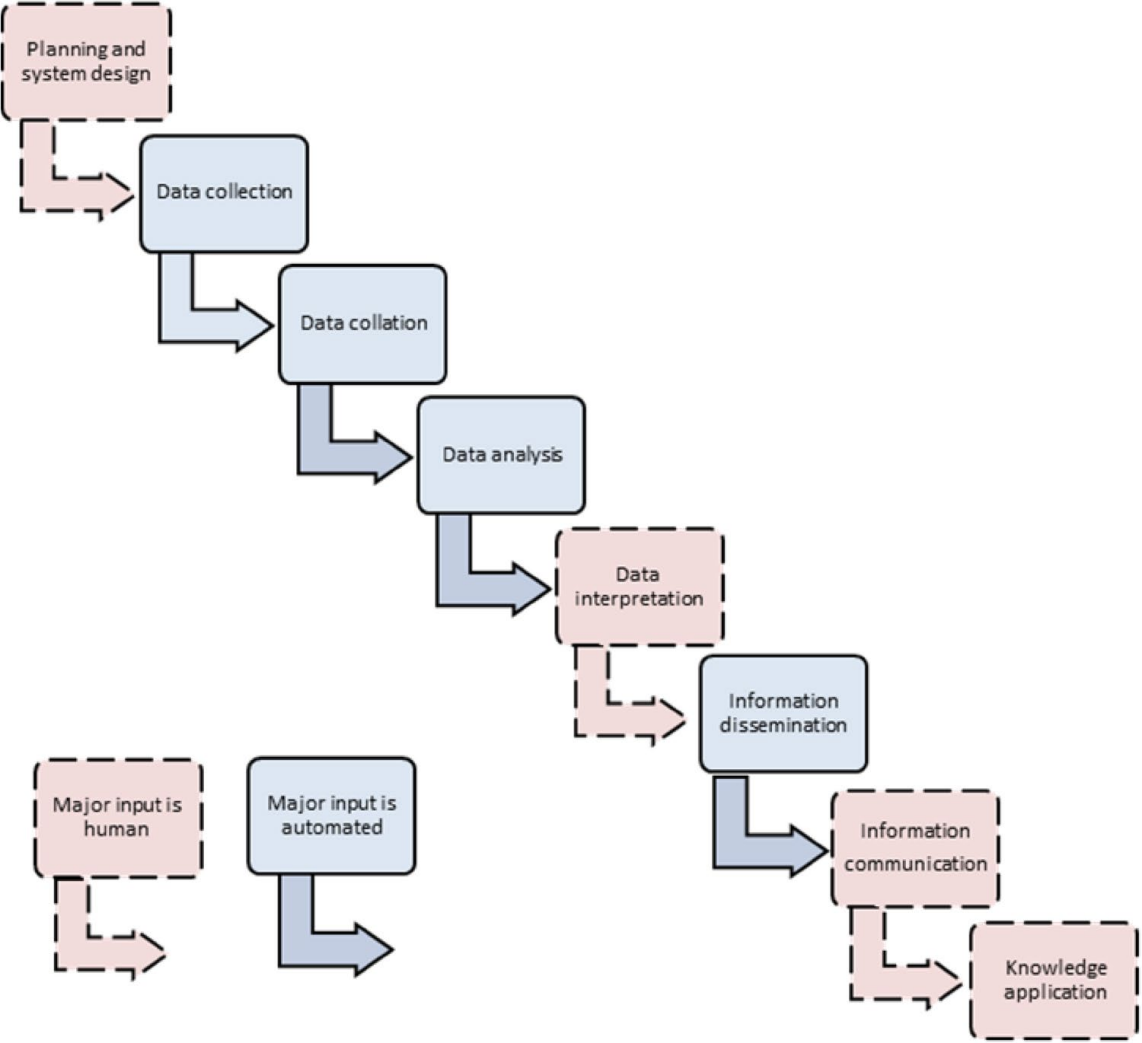
The optimal balance of human and automated inputs into nutrition surveillance activities, adapted from Thacker et al. [134]

Despite the efficiencies that electronic devices might bring, humans will still need to be involved in surveillance and therefore a large source of error will remain, even in high-income countries with sophisticated surveillance systems [133]. Figure 2 illustrates the optimal balance of human and automated inputs into surveillance systems. Developments in technology are likely to help mainly with data collection, collation, analysis and dissemination, but cannot replace humans in planning and system design, and then in interpreting the resulting data.

In particular there have been important recent developments in the use of information and communications technology for data collection [134]. Hand-held electronic devices, including mobile phones to record data, have the potential to improve data quality as described above in the section on data quality. If there is a telephone signal, both raw data and aggregate numbers can be transmitted from hand-held devices and then uploaded to data servers to be merged, or data can be transmitted by Wi-Fi to a local network server to be merged.

However in low-income, rural contexts, telephone signals are often weak or there is no service at all. Thus until basic computer hardware is distributed and its reliability ensured, advantage cannot be taken of the increased speed of data collection offered by adopting mobile phone technology. Most rural community-based health and nutrition centres or monthly growth monitoring events do not have access to a computer, so the nutrition data need to be transferred by paper to the district level health facility and entered there. Often for these paper-based systems, summarised data rather than the complete sets of records are sent to district level, which precludes data quality checks and limits the range of potential data analyses, while bad weather may cause long delays in the collation of the data from remote villages.

As well technical challenges to RTM, such as telephone signals, internet band width and the need for operations research on using these electronic tools and data transmission methods [135], more general challenges include the potentially conflicting priorities of stakeholders from the state, civil society, donors and the private sector. It will be important to ensure that data quality and equity are given precedence over other priorities inherent in the necessary partnerships between public and private stakeholders. Common guidance on quality and equity must be adopted [136].

### Terminology

To aid clarity in future discussions of this topic, we propose definitions for some terms used in this paper. First, it is important to distinguish between the terms ‘surveillance’ as a general activity, and ‘surveillance systems’ as a specific process within this activity. Our proposal is that nutrition surveillance could be simply defined as “The regular and systematic collection of data on nutritional indicators” while a nutrition surveillance system could be defined as: “A system, coordinated by a central institution, that collects primary data that are statistically representative of the population at recurrent intervals on indicators of nutrition and the factors that influence them, for making decisions”. It follows from these definitions that data collected from a health system, including growth monitoring data, could be used for nutrition surveillance, the activity. However the system for the collection, analysis and dissemination of such data would not be classified as a surveillance system because the data were from a health system and therefore not representative. Although above we advocated that secondary data can provide useful indications of local trends in nutrition status, and of national trends between large-scale surveys, we cautioned that such data need to be interpreted together with contextual data, and that if a nutritional problem is identified using secondary data it must verified with data collected using other means. Our proposed definition of a surveillance system may appear to denigrate some existing systems that do not conform to it, however our intention is to emphasise that in low-income contexts, the sole approach that enables effective surveillance by itself is the repeated collection of representative data.

Second it is useful to identify what the term ‘nutrition surveillance system’ encompasses. There are numerous descriptions of nutrition surveillance in the literature and in many cases the activities described were termed surveillance systems. At one end of the continuum lies the system that exists in Bangladesh and which used to exist in Indonesia, which involves repeated rounds of data collection at the same sites, administered centrally by an institution that oversees all activities from data collection and analysis to making policy recommendations. At the other end of the continuum is the approach involving repeated cross-sectional surveys, administered by different agencies, and a central institution compiles the findings to identify trends in the sub-national or national nutrition situation. We suggest that the line between these two models could be drawn based on the existence of a central institution that coordinates the data collection. Thus the systems in South Sudan [57], Ethiopia [137] and Somalia [54] would be termed ‘information systems’ rather than surveillance systems. For each of these three information systems, one of the sources of data is a nutrition surveillance system.

Finally, it is useful to propose a criterion to distinguish surveys from sentinel surveillance because the latter term is not used consistently in the literature, and there is increasing adoption of survey designs which involve small samples that are accepted as being representative of the population from which the samples were drawn. There is overlap between the approaches because in nutritional surveys if the survey site and/or the location of clusters have been purposively sampled, these locations may be called sentinel sites.

As defined by a classic textbook [138], a survey is a “systematic method for gathering information from (a sample of) entities for the purpose of constructing quantitative descriptors of the attributes of the larger population of which the entities are members”. Thus the key feature of a survey is the generalizability of its findings, so the sample is required to be representative of the larger population. The term ‘sentinel’ seems to be used in nutrition surveillance to signify that data collection is from small samples, and therefore the term acts as a warning that the findings are not representative of a larger population but are simply useful for trend analysis.

To enhance consistency in describing activities in different locations, we suggest the distinction between sentinel site surveillance and surveys could be the existence of fixed sites for data collection. We thus define sentinel site surveillance as: “Nutritional assessments at sites that are repeatedly visited”. This definition does not include surveys in which fixed geographic areas are purposively sampled and different clusters within them are chosen at each round, such as ACF’s system in Karamoja [139], or each year such as the Nutrition Surveillance Programme in Ethiopia [95]. It does include systems in which each round of data collection includes the same villages or a purposively selected zone in an urban area, and in which there are new samples of children at each round of data collection, such as the ACF surveillance system in Mathare Valley, Nairobi [50]. It also includes systems in which the same sites are included every round, and new samples of children are selected after a number of rounds. For example, new samples of children are selected each year at the clinics included in the surveillance system in Malawi [87]. To avoid confusion, we suggest that the term “sentinel site surveys” should be avoided (for example ACF’s approach for data collection in Karamoja was described in this way [140]), and that practitioners refer either to surveys, or to sentinel site assessments.

To close this section we return to the classification proposed at the start of the Analysis section, whereby data collection activities were distinguished as generating primary or secondary data. The subsequent review of surveillance activities above will have demonstrated how such a classification is not clear-cut. Most activities designated here as primary involve collection of original data solely for the purposes of surveillance, which are representative of the population as they use probabilistic sampling methods. Most activities designated as secondary involve use of administrative data from health services, so participants are self-selected, and sampling is absent or non-probabilistic. However anomalies which are not consistent with this general pattern exist, including sentinel site surveillance (classified as generating primary data yet sampling is non-probabilistic) and situations in which collection of growth monitoring data through health services has surveillance as the principal objective (classified as generating secondary data even though the data would probably not be generated in the absence of surveillance reporting requirements). No classification scheme can account for every eventuality, and we hope that the scheme proposed above will facilitate discussion and planning of activities.

### Other factors to consider when designing new surveillance activities

We have discussed the technical issues relating to the mechanisms of producing information for nutrition surveillance. When designing new surveillance activities it is necessary but not sufficient to consider these issues as there are also issues relating to political and institutional factors. These have equal or greater bearing on the potential impact of nutrition surveillance than the methods, since there is no point in designing systems to produce information without also ensuring both that these systems will continue for as long there is a need for them, and that the information will be used. In the past, such considerations have at best taken secondary priority to the needs of the methods, or, more often, have simply been ignored. This fact largely explains why, despite great initial enthusiasm and investment, so many systems have been short-lived. We have described the political and institutional factors related to nutrition surveillance elsewhere [3].

## Conclusion

The increased interest in nutrition globally has resulted in high level commitments to reduce and prevent undernutrition. Action to convert these commitments into practice is being hindered by a lack of data. More and effective surveillance of the nutrition situation in countries at every level is needed to support policy and planning, and to provide timely warning of shocks. As the recent Global Nutrition Report identified, rather than necessarily collecting more data, priorities should be to increase the credibility of data currently collected, to focus data collection on a set of core outcome indicators, and to ensure that comparable time series data are collected regularly [18, pp. 113–114].

In this paper, current practices of nutrition surveillance were summarised, and issues with methods identified. To be of value, the resulting information must be credible and so, whichever approach is adopted for surveillance, it must be applied carefully and consistently over time, and there is potential to develop new approaches incorporating aspects of existing methods. It is hoped that insights from this paper relating to methods, together with the political and institutional considerations described in another paper [3], will contribute to the design or amendment of more effective and sustainable activities which will contribute ultimately to the prevention of poor nutrition in low-income countries.

## Footnotes

1 Following registration, CMAM report software can be downloaded from http://www.cmamreport.com/.

2 While the SQUEAC, SLEAC and S3M coverage methods undoubtedly provide useful contextual information for refining service delivery approaches, there are outstanding questions on the validity of the quantitative component of these methods. Several agencies and individuals have voiced a need to compare the sampling approach and analysis to other methods.

3 In fact often the design of the system is such that the findings are statistically representative of the individual site, but the site is acting as a sentinel site for the larger geographical area. For example repeated surveys can provide representative data of a certain slum area, and the slum area acts a sentinel site for the larger urban area. Thus the application of the term ‘sentinel’ depends on the level at which one can validly claim that the findings are statistically representative.

4 The CDC ‘2 surveys’ calculator is helpful as it can be used to examine the effect of different survey designs on the sample size required http://www.cdc.gov/globalhealth/healthprotection/errb/researchandsurvey/calculators.htm.

5 This pilot surveillance system was short-lived. Despite the system having been designed to be as simple as possible, it proved to be difficult to implement in practice.

6 This is true as long as the mean Z score is less than −2. In the exceptional case that the mean Z score is less than −2 (that is, below the threshold to define malnutrition), as variance increases the estimate of prevalence will decrease [38, p. 19]. The proportion of all malnourished children that are severely malnourished also increases.

7 For calculations of necessary sample size, it is recommended to seek guidance from a statistician or a nutritionist with extensive experience in survey design.

8 Anthropometric data from health systems are most useful for detecting trends rather than estimating prevalence rates, so with respect to deriving an overall score to assess data quality, the size of systematic errors is less important for secondary data than for survey data. It is nonetheless important to monitor systematic errors, since changes in size and direction of bias will impact on comparability of data over time.
